# Effects of the primary lung infection on outcomes in patients with severe ARDS treated with ECMO: a retrospective analysis

**DOI:** 10.3389/fmed.2025.1662239

**Published:** 2025-11-05

**Authors:** Martin Mirus, Lars Heubner, Martin Brückner, Thomas Birkner, Andreas Güldner, Axel Rand, Mario Menk, Paul Leon Petrick, Hani Harb, Peter Markus Spieth

**Affiliations:** ^1^Department of Anaesthesiology and Intensive Care Medicine, Faculty of Medicine and University Hospital Carl Gustav Carus, TUD Dresden University of Technology, Dresden, Germany; ^2^Technische Universität Dresden, Dresden, Germany; ^3^Center for Evidence-Based Healthcare, University Hospital Carl Gustav Carus, TUD Dresden University of Technology, Dresden, Germany; ^4^Institute for Medical Microbiology and Virology, Faculty of Medicine and University Hospital Carl Gustav Carus, TUD Dresden University of Technology, Dresden, Germany

**Keywords:** ECMO, critical care, sepsis, infections, ARDS, phenotyping

## Abstract

**Introduction:**

Acute respiratory distress syndrome (ARDS) requiring veno-venous extracorporeal membrane oxygenation (vvECMO) remains associated with high mortality. Whether etiology-based differentiation within infectious ARDS improves prognostic and therapeutic precision remains unclear. This study compared vvECMO-treated ARDS patients with different pulmonary infections to identify clinically relevant etiology-specific differences.

**Methods:**

The retrospective single-center cohort study included adult patients who received vvECMO for severe infectious pulmonary ARDS between 2014 and 2021. Patients were categorized into Covid-19 (*n* = 48) and Non-Covid (*n* = 44). Clinical parameters, disease progression, treatment, and outcomes were compared. Cox and modified Poisson regression analyses identified predictors of ICU mortality.

**Results:**

Non-Covid ARDS patients had greater disease severity at ECMO initiation, although mortality was lower: SOFA score (15.7 vs. 13.7; *p* = 0.003); PRESERVE score (3.73 vs. 2.73; *p* = 0.004). In Covid-19 ARDS, age ≥60 years (RR 1.62), early SOFA score worsening (RR 1.17), new renal replacement therapy (RR 1.60), and septic shock (RR 3.33) were associated with increased mortality, whereas these factors were not predictive in Non-Covid ARDS. Red blood cell transfusion was associated with reduced mortality in both groups (HR 0.96 and 0.95), while fresh frozen plasma transfusion increased mortality exclusively in Covid-19 ARDS (HR 1.09). A rising SOFA score within 5 days after ECMO initiation predicted mortality only in the Covid-19 cohort (RR 1.17).

**Conclusion:**

Even within primary infectious pulmonary ARDS, substantial heterogeneity exists. The underlying infection critically affects the prognostic value of clinical parameters, organ dysfunctions, and scoring systems in vvECMO-treated patients. Considering ARDS etiology may improve risk stratification and guide individualized therapy.

**Trial registration:**

German Clinical Trials Register (DRKS00027856), https://drks.de/search/en/trial/DRKS00027856.

## 1 Introduction

When severe pulmonary acute respiratory distress syndrome (ARDS) due to pulmonary infection requires veno-venous ECMO (vvECMO), additional therapeutic options are limited, and mortality remains high as 34–54% (1, 2). Intensive care therapy focuses mainly on anti-infective and anti-inflammatory components, as well as supportive therapy and the prevention of ventilator-induced lung injury. The current treatments for pulmonary infectious ARDS are usually summarized in ARDS bundles: lung-protective ventilation, prone position, and restrictive fluid therapy (3–7). It remains unclear whether this approach is appropriate for all patients, as ARDS is a syndrome with multidimensional heterogeneity (4, 8, 9). According to current diagnostic criteria, ARDS is simply a description of lung failure without considering its underlying causes (10). This contrasts with evidence showing that the etiology significantly impacts survival (8, 11). At present, differentiation by etiology primarily relies on distinguishing between pulmonary direct and extrapulmonary indirect ARDS (12, 13). Recent research has attempted to further differentiate the heterogeneity of ARDS, i.e., independent of etiology. This has led to the description of many subphenotypes in ARDS patients (14). In some cases, new subphenotypes are defined based on the progression profiles of laboratory biomarkers. Latent class model analyses (15) and machine learning methods are used for correlations of biomarkers in large ARDS datasets and to define ARDS subphenotypes (16). However, the path to translation into clinical routine remains unclear (15). Thus, there are actually two major obstacles to a more differentiated approach to the heterogeneous clinical picture of ARDS in daily clinical practice: Currently, there is no uniform definition of a subphenotype of ARDS, and the relevance of the described subphenotypes is unclear (9, 13, 17). Many recent efforts in subphenotyping still maintain the distinction between intrapulmonary and extrapulmonary ARDS (18–20). This approach is debatable, as the causes of primary intrapulmonary infectious ARDS encompass a broad spectrum, including viruses, bacteria, and fungi. The significant variations within intrapulmonary ARDS based on the infectious agent suggest that a more nuanced understanding of primary intrapulmonary infectious ARDS could be beneficial.

## 2 Materials and methods

### 2.1 Objectives

This study aimed to explore the extent to which a more detailed classification of primary intrapulmonary ARDS is feasible in clinical practice. It focused on patients with severe infectious ARDS caused by direct intrapulmonary infections requiring vvECMO therapy. The objective was to identify etiology-based differences that may are clinically relevant for the prognostic and therapeutic management of severe ARDS.

### 2.2 Study design and setting

Two groups of patients with different pulmonary infections leading to severe infectious ARDS were compared: Non-Covid ARDS ECMO patients and Covid-19 ARDS ECMO patients. A retrospective, single-center study was conducted to evaluate potential differences between these two ARDS groups. All patients were treated according to the standard operating procedures of the Department of Anesthesiology, University Hospital Dresden, Germany, a tertiary care center specializing in ARDS and ECMO treatment for severe lung failure. This publication follows the STROBE cohort guidelines (21). An ethics vote from the relevant committee is available (BO-EK-374072021). The study is registered with the German Clinical Trials Register (DRKS00027856), https://drks.de/search/en/trial/DRKS00027856.

### 2.3 Eligibility criteria

All ECMO cases at the University Hospital Dresden, Germany, from 2014 to 2021 were reviewed via the patient data management system of the intensive care unit (ICU). The medical records were reviewed by two intensivists experienced in the care of critically ill patients who were not involved in further analysis. The inclusion criteria were as follows: age ≥18 years and receiving vvECMO therapy for pulmonary infectious ARDS. To be assigned to the Non-Covid group (NoCov group), both intensivists had to independently classify the case as primary pulmonary infectious ARDS. Cases with no identified pathogen remained eligible for the Non-Covid cohort if both independent intensivists confirmed a pneumonia as cause of ARDS based on criteria according to national pneumonia guidelines (22), i.e., clinical, laboratory, and radiographic evidence, and no alternative non-pulmonary ARDS trigger was present. By contrast, cases labelled ‘ARDS without pulmonary infection’ were excluded because they did not meet pneumonia criteria or had an extrapulmonary origin of ARDS. To be assigned to the Covid group (Cov group), evidence of Covid-19 infection had to be demonstrated by a positive polymerase chain reaction (PCR) test. A lack of pathogen detection was not an exclusion criterion for the NoCov group if the criteria for pneumonia were met (22). To focus on patients with typical infectious pneumonia leading to ARDS, those with ARDS caused by aspiration, drowning, vasculitis, hospital-acquired pneumonia, or fungal infections were excluded. Additionally, patients with ARDS resulting from non-pulmonary causes, such as trauma or non-pulmonary sepsis, were not included.

### 2.4 Variables

The analysis compared Covid-19 and Non-Covid ARDS patients regarding patient characteristics, disease progression, treatment, and outcomes. Each group was analyzed both between and within cohorts by comparing survivors and non-survivors. Short-term mortality was defined as death during the ICU stay. Furthermore, the prognostic impact of clinical and therapeutic parameters was evaluated and contrasted between the two groups. Data were derived from the patient data management system of the ICU.

### 2.5 Statistical analysis

Comparisons between and within ARDS groups were conducted using t-tests or unpaired Wilcoxon tests for continuous variables, while categorical variables were compared using chi-square tests. Two methods were employed to evaluate the impact of parameters on outcomes. First, hazard ratios were calculated using Cox regression analysis. Second, modified Poisson regression analysis was applied to estimate the relative risk of death, as described previously (23, 24). The precision of the relative risk (RR) estimates was expressed using 95% confidence intervals. A *p*-value ≤ 0.05 was considered statistically significant. No adjustments were made for multiple comparisons. All statistical analyses were performed using R statistical software.

## 3 Results

**Figure 1:**
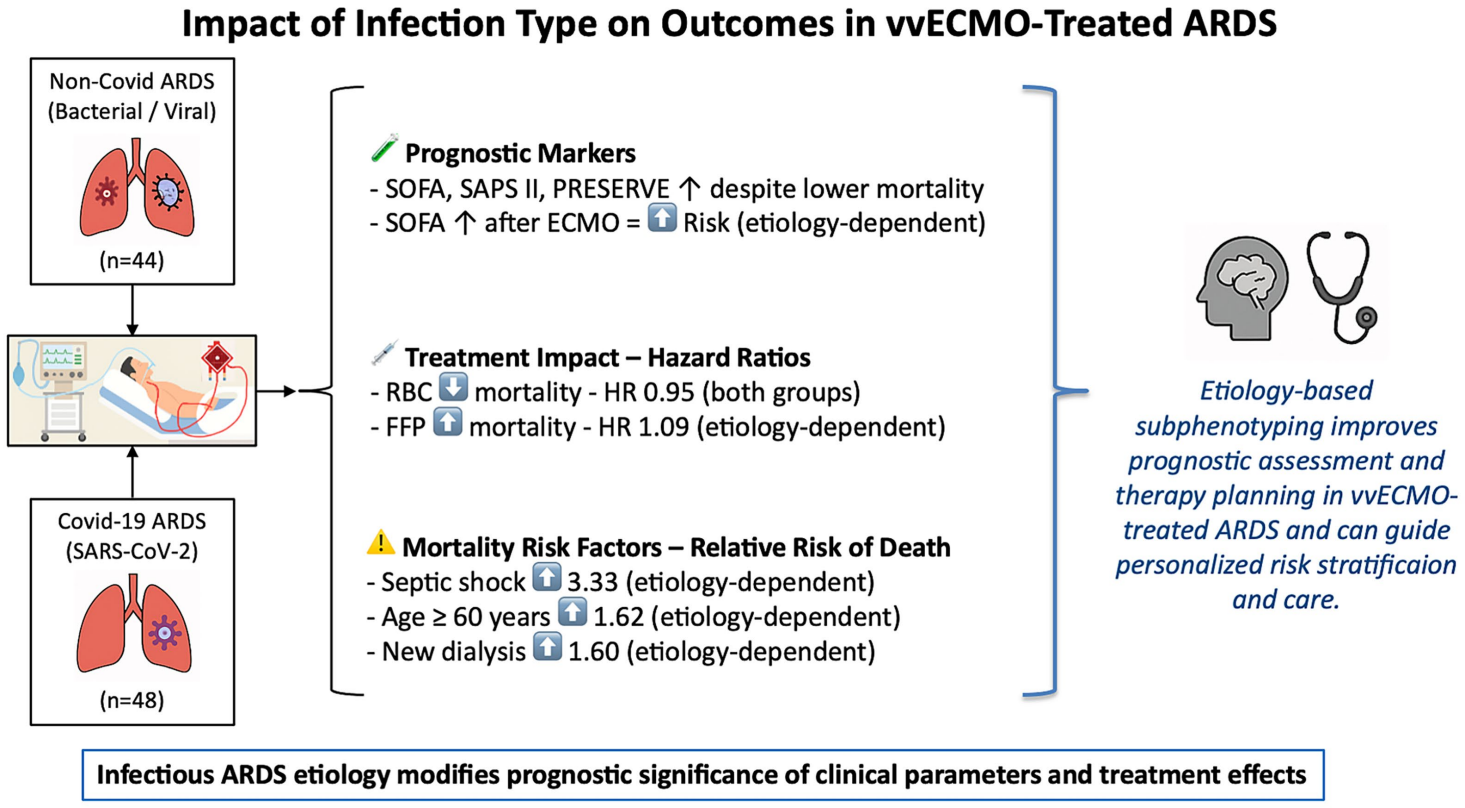
Graphical abstract illustrating the impact of ARDS etiology on prognosis and treatment effects in vvECMO. This figure summarizes the findings of a comparative analysis of Covid-19 and Non-Covid-19 ARDS patients treated with venovenous extracorporeal membrane oxygenation (vvECMO). It highlights key prognostic markers and treatment-associated risk modifiers, such as SOFA score dynamics, transfusion effects, and the etiology-dependent significance of septic shock, age, and renal failure. The results support the concept that infectious etiology influences both outcome prediction and therapeutic response, underlining the value of etiology-based subphenotyping for personalized care in ECMO-treated ARDS. ARDS, Acute Respiratory Distress Syndrome; HR, Hazard ratio; SOFA, Sepsis-related organ failure assessment score; SAPS II, Simplified Acute Physiology Score II; PRESERVE, prediction of death in severe ARDS using the vvECMO score; ECMO, extracorporeal membrane oxygenation; RBC, packed red blood cells; FFP, fresh frozen plasma.

As illustrated in Figure 1, the study design and principal outcomes are summarized in the form of a graphical abstract.

### 3.1 Data extraction and patients

**Figure 2:**
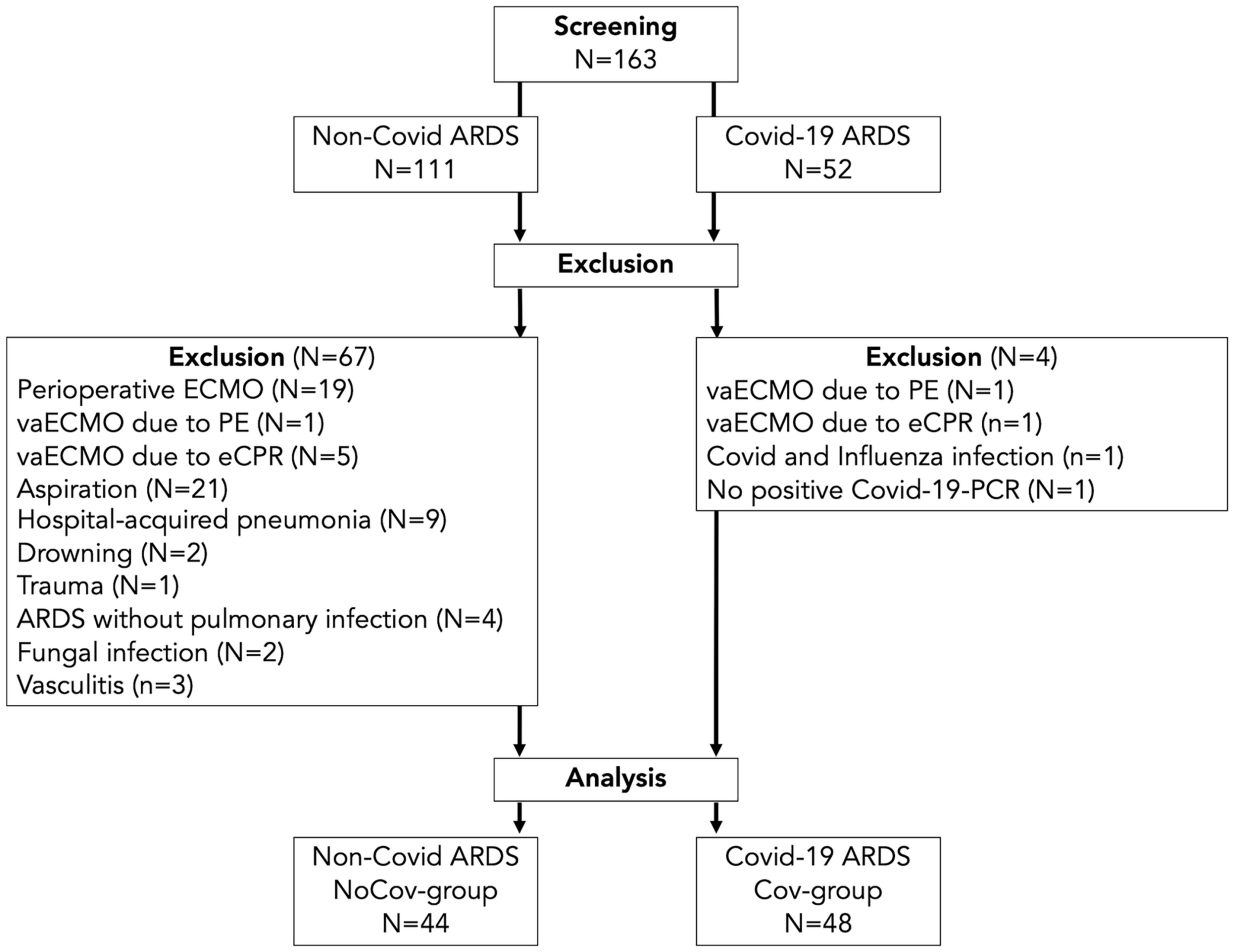
Flow chart of the patient population. In the Cov group, four patients were excluded: two with vaECMO for pulmonary embolism or eCPR, one with synchronous infection with Covid-19 and influenza, and one with missing detection via PCR. In the NoCov group, 67 patients were excluded: six patients with vaECMO for pulmonary embolism or eCPR; 19 patients with perioperative ECMO for planned surgery; 32 patients with aspiration, hospital-acquired pneumonia and drowning; one patient with ECMO for lung trauma; four patients without pulmonary infection; two patients with fungal infection; and three patients with vasculitis. *N* = 48 patients with Covid-19 ARDS and *N* = 44 patients without Covid-19 ARDS were included in the analyses. vaECMO, veno-arterial ECMO; vvECMO, veno-venous ECMO; PCR, polymerase chain reaction; PE, pulmonary embolism; eCPR, extracorporeal cardiopulmonary resuscitation.

A total of 163 patients who received ECMO therapy between 2014 and 2021 were screened. Seventy-one patients were excluded (Figure 2), *N* = 92 vvECMO patients were included in the analysis. Among these patients, 44 had ARDS due to pulmonary infection other than Covid-19 (NoCov group), and 48 had Covid-19 ARDS (Cov group).

### 3.2 Patient characteristics

#### 3.2.1 Comparison between the two ARDS groups

Pre-ARDS patient characteristics did not differ significantly between the two infectious ARDS groups with respect to sex, age, obesity, or antiplatelet therapy (Table 1). The NoCov group had a greater Charlson Comorbidity Index (CCI), was less likely to have cardiovascular disease (CVD), and was more likely to abuse nicotine (Table 1).

**Table 1:** Comparison of pre-ARDS patient characteristics between the two infectious ARDS groups.

Patient characteristic	Non-Covid ARDS	Covid-19 ARDS	*p*
Number of ECMO patients	*N* = 44	*N* = 48	
Female Gender	*N* = 14 (31.8%)	*N* = 9 (18.8%)	0.228
Age, years	58 (10.8)	61 (8.7)	0.197
Obesity	*N* = 24 (34.1%)	*N* = 26 (54.2%)	0.084
Antiplatelet therapy before	*N* = 4 (9.1%)	*N* = 9 (18.8%)	0.326
CCI	2.7 (1.8)	1.3 (1.4)	**<0.001**
Any existing CVD	*N* = 27 (61.4%)	*N* = 42 (87.5%)	**0.008**
Nicotine abuse	*N* = 11 (25%)	*N* = 1 (2.1%)	**0.003**
BMI, kg/m^2^	31 (10.6)	32 (8.1)	**0.028**

Obesity was defined as a BMI≥ 30 kg/m^2^. ECMO, extracorporeal membrane oxygenation; CCI, Charlson Comorbidity Index; BMI, body mass index; CVD, cardiovascular disease (hypertension, chronic heart failure, arrhythmia, coronary heart disease). Unless otherwise noted, the data are presented as the means (SDs). Unpaired two-sample Wilcoxon or chi-square tests were used. Statistical significance (*p*<0.05) is indicated in bold values.

#### 3.2.2 Non-survivors

In the NoCov group, 55% of patients died; in the Cov group, 69% of patients died. Patients who died in the Cov group were significantly older (63 vs. 56 years, *p* = 0.027) than those who survived. In the NoCov group, age was not significantly different between deceased patients and surviving patients (61 vs. 56 years, *p* = 0.125). Comparisons between deceased and surviving patients within the two infectious ARDS groups revealed no significant differences regarding the following pre-ARDS characteristics: sex, obesity, antiplatelet therapy, CCI, CVD, nicotine abuse and BMI (Table 2).

**Table 2:** Comparison of pre-ARDS patient characteristics between non-surviving and surviving patients in both ARDS groups.

Patient characteristic	Non-Covid ARDS			Covid-19 ARDS		
	Not survived	Survived	*p*	Not survived	Survived	*p*
Number of patients	*N* = 24 (54.5%)	*N* = 20 (45.5%)		*N* = 33 (68.8%)	*N* = 15 (31.3%)	
Female sex	*N* = 5 (20.8%)	*N* = 9 (45%)	0.165	*N* = 4 (12.1%)	*N* = 5 (33.3%)	0.178
Male sex	*N* = 19 (79.2%)	*N* = 11 (55%)		*N* = 29 (87.9%)	*N* = 10 (66.7%)	
Age, years	61 (10.5)	56 (10.7)	0.125	63 (7.4)	56 (9.8)	**0.027**
Obesity	*N* = 7 (29.2%)	*N* = 8 (40%)	0.663	*N* = 16 (48.5%)	*N* = 10 (66.7%)	0.39
Antiplatelet drugs before	*N* = 3 (12.5%)	*N* = 1 (5%)	0.67	*N* = 6 (18.2%)	*N* = 3 (20%)	1
CCI	2.9 (1.6)	2.4 (2)	0.275	1.2 (1.4)	1.4 (1.4)	0.569
Any existing CVD	*N* = 15 (62.5%)	*N* = 12 (60%)	1	*N* = 28 (84.8%)	*N* = 14 (93.3%)	0.724
Nicotine abuse	*N* = 6 (25%)	*N* = 5 (25%)	1	*N* = 0	*N* = 1 (6.7%)	0.683
BMI, kg/m^2^	29 (6.5)	33 (14)	0.785	32 (8.7)	33 (7)	0.482

CCI, Charlson Comorbidity Index; BMI, body mass index; CVD, cardiovascular disease (hypertension, chronic heart failure, arrhythmia, coronary heart disease). Unless otherwise noted, the data are presented as the means (SDs). Unpaired two-sample Wilcoxon or chi-square test. Statistical significance (*p*<0.05) is indicated in bold values.

### 3.3 Course of the disease

#### 3.3.1 Comparison between the two ARDS groups

Patients in the NoCov group had fewer days in the hospital before ECMO therapy, higher SAPS II scores and higher SOFA scores at ICU admission, higher SOFA scores at ECMO initiation, and lower SOFA scores at ICU discharge (Table 3). The PRESERVE (predicting death for severe ARDS on vvECMO) score at ECMO initiation was higher in the NoCov group (Table 3). In summary, despite greater disease severity and organ dysfunction reflected by established ICU scores, Non-Covid ARDS patients had lower mortality compared to those with Covid-19 ARDS.

**Table 3:** Comparison of ARDS course parameters between the two infectious ARDS groups.

ARDS course parameter	Non-Covid ARDS	Covid-19 ARDS	*p*
Number of patients	*N* = 44	*N* = 48	
Days in hospital before ECMO	8.3 (9.3)	10.9 (5.9)	**0.005**
Days intubated before ECMO	4.8 (6.5)	5.7 (5)	0.129
SAPS II at ICU admission	47.7 (15.0)	40.6 (9.7)	**0.013**
SOFA at ICU admission	15.5 (3.3)	12.6 (3.0)	**<0.001**
SOFA at ECMO-start	15.7 (3.1) (missing *N* = 1)	13.7 (2.6)	**0.003**
SOFA 5 days after ECMO-start	14.4 (3.80)	14.0 (3.41)	0.62
SOFA at discharge	5.8 (5.67)	8.6 (3.54)	**0.048**
Lung Injury Score at ECMO-start	3.25 (0.51)	3.54 (0.32) (missing *N* = 1)	**0.002**
PRESERVE at ECMO-start	3.73 (1.63) 4 (median) (missing *N* = 4)	2.73 (1.38) 3 (median)	**0.004**
Infectious source			
Bacterial	*N* = 14 (31.8%)	*N* = 48 (100%)	**<0.001**
Viral	*N* = 15 (34.1%)		
No detection	*N* = 15 (34.1%)		
Coinfection	*N* = 32 (73%)	*N* = 44 (92%)	**0.034**
Platelets at ECMO-start (Gpt/l)	187 (138)	233 (116)	**0.026**
Fibrinogen at ECMO-start (g/l)	5.8 (2.0)	7.2 (2.0)	**<0.001**
Fibrinogen max (g/l)	7.7 (1.7)	8.7 (1.6)	**0.002**
DD max (ng/ml)	3,470 (1090)	14,400 (8940)	**<0.001**
PCT at ICU admission (ng/ml)	46.8 (91.4)	4.6 (8.2)	**0.006**
Urea at ECMO-start (mmol/l)	13.8 (7.61)	14.0 (7.56)	0.793
Bili max (μ mol/l)	77.4 (111)	119 (113)	**0.015**
Thromboses			
Deep vein thrombosis	*N* = 0	*N* = 14 (29%)	**0.004**
Pulmonary embolism	*N* = 3 (7%)	*N* = 17 (35%)	**0.002**
Non	*N* = 40 (91%)	*N* = 29 (60%)	**0.002**

SAPS II, simplified acute physiology score II; SOFA, sepsis-related organ failure assessment score; PRESET, prediction of survival with the ECMO therapy score; RESP, respiratory ECMO survival prediction score; PRESERVE, prediction of death for severe ARDS with the vvECMO score; DD, D-dimer; PCT, procalcitonin; CRP, C-reactive protein; Crea, creatinine; Bili, total bilirubin; unless otherwise noted, mean (SD). Unpaired two-sample Wilcoxon or chi-square test. Statistical significance (*p*<0.05) is indicated in bold values.

#### 3.3.2 Non-survivors

In the NoCov group, 55% of patients died; in the Cov group, 69% of patients died. Non-survivors in the NoCov group had significantly more hospital days before ECMO initiation and a higher PRESERVE score (Table 4). Other parameters describing the course of ARDS were not significantly different between deceased and surviving patients in the NoCov group (Table 4). Non-survivors in the Cov group had a higher SAPS II score at ICU admission, a higher SOFA score 5 days after ECMO initiation, a higher PRESERVE score, and lower platelets (Table 4). Comparison of the ARDS course between deceased and surviving patients within the two infectious ARDS groups revealed no significant differences between groups with respect to intubation days before ECMO, SOFA score at ICU admission and before ECMO initiation, coinfections and peak procalcitonin levels (Tables 4; Supplementary Table S4).

**Table 4:** Comparison of the ARDS course between non-surviving and surviving patients in both ARDS groups.

ARDS course parameter	Non-Covid ARDS			Covid-19 ARDS		
	Not survived	Survived	*p*	Not survived	Survived	*p*
Number of patients	*N* = 24 (54.5%)	*N* = 20 (45.5%)		*N* = 33 (68.8%)	*N* = 15 (31.3%)	
Days in hospital before ECMO	10.4 (10.2)	5.9 (7.6)	**0.029**	10.7 (6.2)	11.2 (5.4)	0.824
Days intubated before ECMO	5.3 (6.6)	4.2 (6.5)	0.098	5.9 (4.6)	5.5 (5.9)	0.554
SAPS II at ICU admission	50.6 (14.3)	44.2 (15.6)	0.243	43.6 (9.7)	34.1 (5.6)	**0.001**
SOFA at ECMO-start	16 (2.8)	15.3 (3.3)	0.532	14.2 (2.8)	12.5 (1.9)	0.071
SOFA 5d after ECMO-start	13.8 (3.89)	14.9 (3.74)	0.502	15.4 (3.20)	11.4 (2.06)	**<0.001**
Lung Injury Score at ECMO-start	3.28 (0.569)	3.22 (0.445)	0.45	3.56 (0.33)	3.51 (0.28)	0.38
PRESERVE at ECMO-start	4.38 (1.43) 4 (median)	3.00 (1.56) 3 (median)	**0.01**	3.12 (1.24) 3 (median)	1.87 (1.30) 2 (median)	**0.005**
Coinfection	*N* = 18 (75%)	*N* = 14 (70%)	0.975	*N* = 31 (94%)	*N* = 13 (87%)	0.778
Platelets ECMO-start (Gpt/l)	165 (120)	213 (156)	0.334	201 (100)	302 (120)	0.008
Urea at ECMO-start (mmol/l)	13.9 (7.62)	13.6 (7.80)	0.85	15.6 (8.18)	10.4 (4.40)	**0.018**
Bili max (μ mol/l)	106 (142)	43.2 (36.4)	0.107	145 (116)	61.2 (82.3)	**0.002**
Thromboses						
DVT	*N* = 0	*N* = 0	0.87	*N* = 12 (36%)	*N* = 2 (13%)	**0.013**
PE	*N* = 1 (4%)	*N* = 2 (10%)	1	*N* = 13 (39%)	*N* = 4 (27%)	0.597
Non	*N* = 22 (92%)	*N* = 18 (90%)		*N* = 20 (61%)	*N* = 9 (60%)	1

SAPS II, simplified acute physiology score II; SOFA, sepsis-related organ failure assessment score; PRESET, prediction of survival with the ECMO therapy score; RESP, respiratory ECMO survival prediction score; PRESERVE, prediction of death for severe ARDS with the vvECMO score; DD, D-dimer; PCT, procalcitonin; CRP, C-reactive protein; Crea, creatinine; Bili, total bilirubin; GGT, serum gamma-glutamyl transferase; unless otherwise noted, mean (SD). Unpaired two-sample Wilcoxon or chi-square test. Statistical significance (*p*<0.05) is indicated in bold values.

### 3.4 Treatment characteristics

#### 3.4.1 Comparison between the two ARDS groups

Patients in the NoCov group had fewer prone therapy sessions, more days intubated to tracheostomy, lower maximal ECMO blood flow, and lower maximal ECMO sweep gas flow. They exhibited lower ventilation pressures before ECMO and 24 h after ECMO initiation (Table 5). Dexamethasone therapy was less frequently used in the NoCov group, whereas the use of prednisolone and other steroids revealed no differences (Table 5). A comparison of other parameters related to ARDS therapy is shown in Supplementary Table S5.

**Table 5:** Comparison of ARDS therapy between the two infectious ARDS groups.

Therapy parameter	Non-Covid ARDS	Covid-19 ARDS	*p*
Number of patients	*N* = 44	*N* = 48	
Proning	*N* = 36 (81.8%)	*N* = 42 (87.5%)	0.64
Number of pronings	4 (3.8)	6 (4.1)	**0.007**
Pronings before ECMO	2 (1.8)	3 (2.8)	**0.011**
NO therapy	*N* = 13 (29.5%)	*N* = 30 (62.5%)	**0.003**
Tracheostomy	*N* = 20 (45.5%)	*N* = 27 (56.3%)	0.409
Days intubated till tracheostomy	15.8 (7)	10.9 (6.1)	**0.018**
ECMO BF max (l/min)	4.2 (1.1)	5.1 (2.4)	**0.01**
Sweep max (l/min)	5.1 (2)	7 (2.5)	**<0.001**
Ppeak 24 h after ECMO start (mbar)	25.7 (3.5)	28.0 (2.8)	**0.003**
Ppeak before ECMO-end (mbar)	21.5 (4.5)	26.6 (3.2)	**0.006**
Ppeak 24 h after ECMO-end (mbar)	22.7 (4.5)	28.3 (6.2)	**0.026**
PEEP 24 h after ECMO start (mbar)	11.3 (3.8)	13.8 (1.5)	**0.002**
VT (ml/kg IBW) 24 h after ECMO-start	4.06 (1.55)	4.86 (1.50)	**0.035**
Steroids			
Dexamethasone	*N* = 0	*N* = 44 (92%)	**<0.001**
Prednisolone	*N* = 18 (41%)	*N* = 22 (46%)	0.791
Other	*N* = 6 (14%)	*N* = 10 (21%)	0.526
RBCs/day stay	0.841 (0.991)	0.835 (0.677)	0.306
FFPs	6.93 (13.7)	3.02 (4.78)	0.39

NO, nitrous oxide; BF, blood flow; Ppeak, peak inspiratory pressure; PEEP, positive end-expiratory pressure; VT, tidal volume; IBW, ideal body weight; RBC, packed red blood cells; FFP, fresh frozen plasma. Unless otherwise noted, the data are presented as the means (SDs). Unpaired two-sample Wilcoxon or chi-square test. Statistical significance (*p*<0.05) is indicated in bold values.

#### 3.4.2 Non-survivors

In the NoCov group, 55% of patients died; in the Cov group, 69% of patients died. Non-survivors in the NoCov group had more pronings before the initiation of ECMO therapy. The total number of pronings was not different between deceased and surviving patients. Non-survivors in the NoCov group received fewer tracheostomies, had higher maximum ECMO blood flow, higher maximum ECMO sweep gas flow, lower tidal volumes 24 h after ECMO, and needed more packed red blood cells (Table 6). Non-survivors in the Cov group received fewer tracheostomies, higher maximum ECMO blood flow (Table 6), higher maximum ECMO sweep gas flow, and more fresh frozen plasma transfusions (Table 6). There was no difference in the number of pronings before ECMO initiation or tidal volume 24 h after ECMO initiation. A comparison of other parameters between deceased and surviving patients receiving ARDS therapy is shown in Supplementary Table S6.

**Table 6:** Comparison of ARDS therapy between non-surviving and surviving patients in both ARDS groups.

Therapy parameter	Non-Covid ARDS			Covid-19 ARDS		
	Not survived	Survived	*p*	Not survived	Survived	*p*
Number of patients	*N* = 24 (54.5%)	*N* = 20 (45.5%)		*N* = 33 (68.8%)	*N* = 15 (31.3%)	
Proning	*N* = 19 (79%)	*N* = 17 (85%)	0.915	*N* = 29 (88%)	*N* = 13 (87%)	1
Number of pronings	4.74 (4.71)	3.18 (2.19)	0.403	6 (3.38)	6.23 (5.49)	0.623
Pronings before ECMO	2.47 (2.09)	0.76 (0.66)	**0.003**	3.34 (2.94)	2.77 (2.62)	0.6
NO therapy	*N* = 8 (33%)	*N* = 5 (25%)	0.786	*N* = 21 (64%)	*N* = 9 (60%)	1
Tracheostomy	*N* = 7 (29%)	*N* = 13 (65%)	**0.038**	*N* = 14 (42%)	*N* = 13 (87%)	**0.011**
Days intubated till tracheostomy	16.0 (6.30)	15.6 (7.63)	0.937	9.29 (4.73)	12.7 (7.11)	0.214
ECMO BF max (l/min)	4.57 (0.864)	3.74 (1.17)	**0.013**	5.53 (2.68)	4.16 (0.968)	**<0.001**
Sweep max (l/min)	5.80 (1.94)	4.27 (1.87)	**0.008**	7.79 (2.11)	5.17 (2.37)	**<0.001**
Ppeak 24 h after ECMO-end (mbar)	24.0	22.6 (4.57)	0.714	28.0	28.3 (6.74)	1
VT (ml/kg IBW) 24 h after ECMO-start	3.40 (1.53)	4.76 (1.25)	**0.006**	4.59 (1.52)	5.40 (1.33)	0.096
Steroids						
Dexa	*N* = 0	*N* = 0	**0.023**	*N* = 30 (91%)	*N* = 14 (93%)	1
Predni	*N* = 14 (58%)	*N* = 4 (20%)	0.049	*N* = 14 (42%)	*N* = 8 (53%)	0.696
Other	*N* = 6 (25%)	*N* = 0		*N* = 9 (27%)	*N* = 1 (7%)	0.213
RBCs/day stay	1.18 (1.23)	0.430 (0.230)	**0.023**	0.970 (0.728)	0.539 (0.437)	**0.03**
FFPs	9.04 (16.8)	4.40 (8.47)	0.158	4.30 (5.29)	0.200 (0.561)	**0.009**

NO, nitrous oxide; BF, blood flow; Ppeak, peak inspiratory pressure; PEEP, positive end-expiratory pressure; VT, tidal volume; IBW, ideal body weight; Dexa, dexamethasone; Predni, prednisolone; RBC, packed red blood cells; FFP, fresh frozen plasma. Unless otherwise noted, the data are presented as the means (SDs). Unpaired two-sample Wilcoxon or chi-square test. Statistical significance (*p*<0.05) is indicated in bold values.

### 3.5 Parameters influencing hazard ratios in both ARDS groups

#### 3.5.1 Regarding patient characteristics

In neither the NoCov nor the Cov group did any pre-ARDS patient characteristics significantly influence the hazard ratios (Table 7).

**Table 7:** Comparison of both infectious ARDS groups regarding parameters influencing hazard ratios.

Parameter	Non-Covid ARDS	Covid-19 ARDS
Number of not survived patients (%)	*N* = 24 (54.5%)	*N* = 33 (68.8%)
Pre-ARDS patient characteristics		
Age≥ 60 years	1.33 (0.59, 3.00; 0.5)	1.90 (0.88, 4.08; 0.1)
Female sex	0.59 (0.22, 1.59; 0.3)	0.47 (0.17, 1.36; 0.2)
BMI ≥ 35 kg/m^2^	0.54 (0.15, 1.93; 0.3)	0.95 (0.46, 1.96; 0.9)
ARDS-course		
SAPS II at ICU	1.03 (1.00, 1.06; 0.074)	1.06 (1.03, 1.10; **<0.001**)
SOFA at ICU	1.11 (0.97, 1.26; 0.12)	1.25 (1.11, 1.40; **<0.001**)
SOFA 5d after ECMO-start	1.03 (1.02, 1.04; **<0.001**)	1.03 (1.02, 1.05; **<0.001**)
GGT max (μ mol/s*l)	0.92 (0.86, 0.98; **0.013**)	0.98 (0.95, 1.00; 0.073)
Septic shock	2.80 (0.66, 12.0; 0.2)	5.26 (1.59, 17.4; **0.007**)
SOFA trend (first 5 days of ECMO)	0.92 (0.75, 1.13; 0.4)	1.22 (1.01, 1.47; **0.039**)
ARDS-therapy		
Proning	0.32 (0.11, 0.9; **0.031**)	0.69 (0.24, 1.97; 0.5)
Tracheostomy	0.27 (0.11, 1.13; **0.004**)	0.31 (0.15, 0.65; **0.002**)
RBCs	0.96 (0.92, 1.00; **0.032**)	0.95 (0.91, 0.99; **0.020**)
FFPs	1.02 (0.99, 1.05; 0.14)	1.09 (1.03, 1.16; **0.006**)

Univariate Cox regression analysis was performed for both infectious ARDS groups. The analysis was carried out for: pre-ARDS patient characteristics, ARDS course, and ARDS therapy. Depending on the type of infectious ARDS, different parameters significantly influence hazard ratios. BMI, body mass index; SAPS II, simplified acute physiology score; SOFA, sepsis-related organ failure assessment score; ECMO, extracorporeal membrane oxygenation; GGT, gamma-glutamyltransferase; RBC, red blood cell; FFP, fresh frozen plasma. Hazard ratios (95% confidence intervals; p values). Statistical significance (*p*<0.05) is indicated in bold values.

#### 3.5.2 Regarding the course of ARDS

In the NoCov group, univariate Cox regression revealed that SOFA score 5 days after ECMO initiation significantly influenced the hazard ratio (HR 1.03) (Table 7). In the Cov group, univariate Cox regression revealed a significant increase in the hazard ratio according to SOFA and SAPS II score at ICU admission (HR 1.25 and 1.06) and the occurrence of septic shock in the course of ARDS (HR 5.26) (Table 7). An increase in the SOFA score from ECMO initiation to day five was associated with a higher hazard ratio (HR 1.22), whereas this association was not statistically significant in the NoCov group (Table 7).

#### 3.5.3 Regarding ARDS therapy

In the NoCov group, proning therapy (HR 0.32), tracheostomy (HR 0.27), and a higher number of red blood cell (RBC) transfusions (HR 0.96) were each associated with a significant reduction in the hazard ratios (Table 7). In the Cov group, both tracheostomy (HR 0.31) and RBC transfusion (HR 0.95) were similarly associated with reduced mortality. In contrast, transfusion of fresh frozen plasma (FFP) was associated with an increased hazard ratio (HR 1.09) (Table 7).

### 3.6 Parameters influencing the relative risk of death in both ARDS groups

#### 3.6.1 Regarding pre-ARDS characteristics

In the Cov group, age ≥60 years (RR 1.62) significantly increased the relative risk of death, not in the NoCov group (Table 8).

**Table 8:** Comparison of the two infectious ARDS groups with respect to parameters influencing the relative risk of death.

Parameter	Non-Covid ARDS	Covid-19 ARDS
Number of not survived patients (%)	*N* = 24 (54.5%)	*N* = 33 (68.8%)
Pre-ARDS patient characteristics^*^		
Age≥ 60	1.35	1.62 (**0.033**)
Female sex	0.58	0.61
BMI ≥ 35 kg/m^2^	0.77	0.98
ARDS-course		
SAPS II at ICU	1.01	1.03 (**0.001**)
SOFA at ICU	1.02	1.07 (**0.008**)
SOFA trend (first 5 days of ECMO)	0.92	1.17 (**0.001**)
Septic shock	2.08	3.33 (**0.017**)
ARDS-therapy		
Tracheostomy	0.49 (**0.034**)	0.57
New CRRT	1.06	1.60 (**0.018**)
RBC transfusion	0.9992	0.99
FFP transfusion	1.01 (0.013)	1.05 (**0.0002**)

Modified Poisson regression analysis for both infectious ARDS groups. The analysis was carried out for: pre-ARDS patient characteristics, ARDS course, and ARDS therapy. Depending on the cause of ARDS, different parameters significantly influence the relative risk of death. BMI, body mass index; SAPS II, simplified acute physiology score; SOFA, sepsis-related organ failure assessment score; ECMO, extracorporeal membrane oxygenation; CRRT, continuous renal replacement therapy; RBC, red blood cell; FFP, fresh frozen plasma. Unless otherwise indicated, univariate modified Poisson regression analyses were performed. Relative risk (*p* value).

* Multivariate Poisson regression. Statistical significance (*p*<0.05) is indicated in bold values.

#### 3.6.2 Regarding the course of ARDS

**Figure 3:**
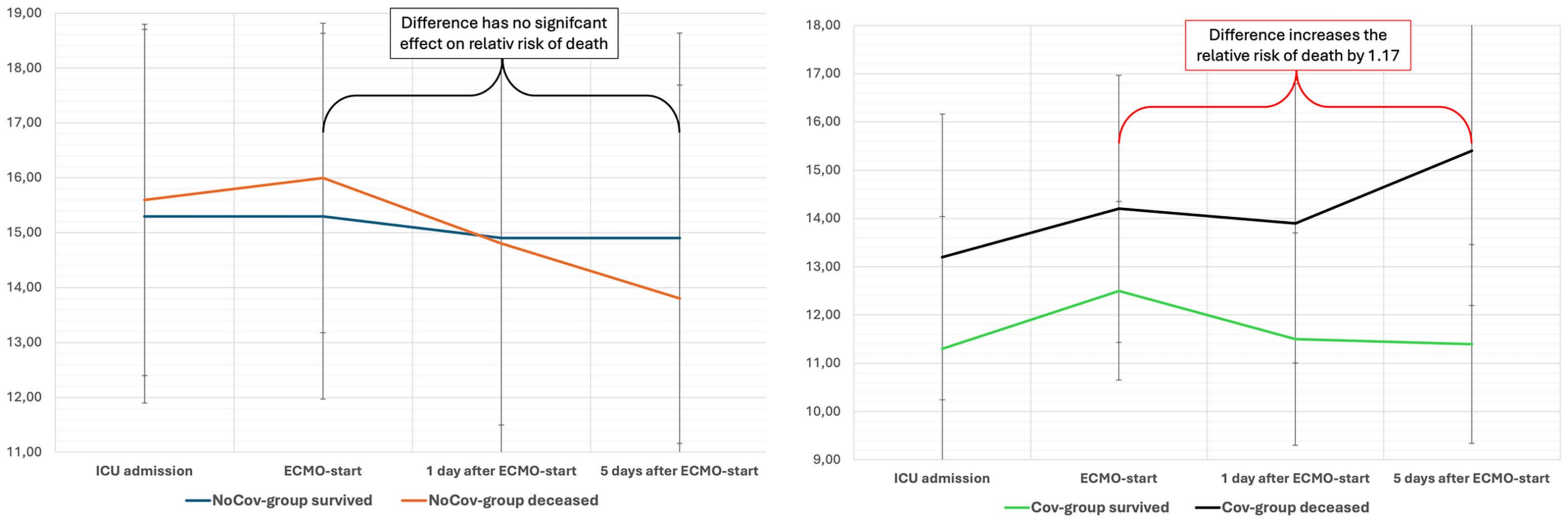
The evolution of the SOFA score in both ARDS cohorts after ECMO initiation. The impact of ARDS etiology on outcome prediction is illustrated by the evolution of the SOFA score within 5 days of ECMO initiation. In this study, changes in SOFA scores during the early ECMO course were analyzed to assess their association with mortality. In the Non-Covid ARDS group (left panel), non-survivors showed a nonsignificant trend toward a decrease in SOFA score. In contrast, an increase in SOFA score in the Covid-19 ARDS group (right panel) was significantly associated with a higher relative risk of death, highlighting the prognostic value of dynamic organ dysfunction—depending on ARDS etiology. The mean SOFA score and SD are shown. ICU, intensive care unit; ECMO, extracorporeal membrane oxygenation.

In the Cov group, a higher SAPS II score (RR 1.03) and higher SOFA score (RR 1.07) at ICU admission increased the relative risk of death. An increase in the SOFA score within the first 5 days of ECMO therapy was associated with an increased relative risk of death in the Cov group (RR 1.17; Table 8; Figure 3). In the Cov group, both the need for new dialysis (RR 1.60) and the occurrence of septic shock (RR 3.33) significantly increased the relative risk of death. None of the above parameters significantly influenced the relative risk of death in the NoCov group (Table 8).

#### 3.6.3 Regarding ARDS therapy

In the NoCov group, tracheostomy was associated with a lower relative risk of death (RR 0.49). This effect was not significant in the Cov group. In the Cov group, fresh frozen plasma transfusion increased the relative risk of death (RR 1.05), whereas it was not significant in the NoCov group (Table 8).

## 4 Discussion

This study demonstrates that the prognostic relevance of individual parameters and new organ dysfunction in patients with severe ARDS on vvECMO critically depends on the underlying cause of ARDS. In Covid-19 ARDS, age (RR 1.62), new need for dialysis (RR 1.60), and the development of septic shock (RR 3.33) significantly increased the relative risk of death. These associations were not observed in Non-Covid ARDS. Conversely, transfusion of red blood cells was associated with reduced mortality in both ARDS groups, while transfusion of fresh frozen plasma was associated with increased mortality only in Covid-19 ARDS.

These findings emphasize the importance of ARDS subphenotyping, even in cases of primary infectious pulmonary ARDS. Despite a shared clinical syndrome, Covid-19 and Non-Covid ARDS exhibited distinct prognostic profiles. This suggests that differentiation by ARDS etiology is both feasible in routine clinical settings and clinically meaningful, particularly when it alters the prognostic value of individual risk factors.

While several studies have compared severe Non-Covid ARDS and Covid-19 ARDS with a focus on clinical trajectory (25, 26), only few have specifically addressed this comparison in patients receiving ECMO support (27–29). Moreover, many of these studies included heterogeneous ARDS etiologies, such as trauma-related ARDS, pneumonitis, pancreatitis-associated ARDS, or COPD exacerbations (30). The inclusion of such mixed ARDS entities may obscure important differences and limit the generalizability of findings to direct pulmonary infectious ARDS. In contrast, the present study focused on a clinically homogeneous cohort of patients with severe ARDS of direct infectious pulmonary origin requiring vvECMO. This targeted approach allows for a more nuanced understanding of pathophysiological differences between Covid-19 and Non-Covid ARDS and strengthens the rationale for cause-specific subphenotyping.

### 4.1 Baseline characteristics and risk stratification

The cohort was homogeneous in terms of disease severity and critical care interventions, with all patients requiring invasive ventilation and vvECMO for severe infectious pulmonary ARDS. Among pre-ARDS characteristics, only age was associated with mortality—and only in Covid-19 ARDS patients. In this group, deceased patients were significantly older (63 vs. 56 years), and age ≥60 years was associated with an increased risk of death (RR 1.62). This aligns with findings from Tonetti et al., who reported greater age-related mortality in Covid-19 ARDS ECMO patients compared to Non-Covid patients (31), and extends previous registry data that did not differentiate by ARDS etiology (32).

### 4.2 Course of disease and limitations of established scores

Despite lower mortality in the Non-Covid ARDS group (54.5% vs. 68.8%), severity scores at ECMO initiation—SOFA, SAPS II, and PRESERVE—were paradoxically higher. This counterintuitive finding mirrors observations by Cousin et al. in influenza ARDS (33) and highlights limitations in applying general prognostic scores across heterogeneous ARDS subtypes. Specifically, the PRESERVE score, originally developed in a mixed ARDS cohort, overestimated survival in Non-Covid ARDS (expected 6-month mortality 21% with score 3.7 vs. observed 54.5%).

Importantly, only in Covid-19 ARDS did an increase in SOFA score within 5 days of ECMO initiation correlate with an increased relative risk of death (RR 1.17). Similarly, septic shock significantly worsened prognosis only in this subgroup (RR 3.33). These findings underscore the need to interpret organ dysfunction scores in the context of ARDS etiology.

### 4.3 Therapeutic implications

The effect of therapeutic interventions also varied by ARDS cause. Prone positioning reduced the hazard of death significantly—but only in Non-Covid ARDS (HR 0.32). Likewise, while new-onset dialysis was more frequent in Non-Covid ARDS, only in Covid-19 ARDS did it predict increased mortality (RR 1.60). This challenges the assumption that the development of additional organ failure, such as renal dysfunction requiring CRRT, uniformly indicates poor prognosis in all ARDS subtypes.

### 4.4 Limitations

This study has several limitations. First, it is a single-center retrospective analysis with a relatively small sample size, which limits generalizability and statistical power. Second, treatment strategies for ARDS have evolved over the past 7 years, particularly during the COVID-19 pandemic. While the center’s overall treatment approach remained consistent, new anti-infective agents and anti-inflammatory therapies may have influenced outcomes over time.

Another important limitation is the heterogeneity of pathogens within the Non-Covid ARDS group. Although the study focused exclusively on direct intrapulmonary infectious ARDS to ensure a more homogeneous cohort, this study was not powered for pathogen-specific subgroup analyses. Although bacterial and non-bacterial ARDS represent distinct biological entities, the number of cases within each etiologic category of the Non-Covid cohort was too small to allow for meaningful statistical comparison. For these reasons, no separate analysis of bacterial versus non-bacterial ARDS was performed, as this would risk overinterpretation of small, heterogeneous subgroups. So, pathogen-specific comparisons were not possible. Nonetheless, our findings highlight the potential relevance of etiologic differentiation, which should be addressed in future prospective studies designed and powered for this specific purpose. This limits the ability to distinguish outcome differences based on microbial etiology. Moreover, nosocomial and aspiration pneumonia cases were excluded to maintain consistency with community-acquired infectious ARDS, as these subgroups should be analyzed separately (22). We excluded aspiration ARDS because the primary mechanism is chemical pneumonitis with potential secondary infection rather than primary pneumonia.

Pulmonary coinfections further complicate the interpretation of results. Coinfections were observed in 73% of Non-Covid and 92% of Covid-19 ARDS cases, potentially influencing disease progression and response to treatment. However, their exact impact remains uncertain. While some studies suggest an association with prolonged ICU stay, coinfections do not appear to significantly affect mortality (34).

This study lacked systematic post-discharge follow-up, including long-term survival, pulmonary function, and imaging. Ethical approval and patient consent for such follow-up were beyond the scope of this retrospective analysis. While we report in-hospital mortality, assessment of post-ARDS fibrosis would require prospective studies with standardized longitudinal imaging and structured follow-up protocols.

Future studies should aim to validate these findings in larger, multicenter cohorts and explore the role of pathogen-specific factors and coinfections in shaping the course and outcome of pulmonary infectious ARDS.

## 5 Conclusion

This study highlights the importance of considering ARDS etiology in prognostication and therapeutic decision-making for patients suffering severe ARDS treated with vvECMO. Covid-19 and Non-Covid infectious pulmonary ARDS differ not only in baseline characteristics and disease trajectory but also in the prognostic relevance of organ dysfunction and therapeutic interventions. Established severity scores such as SOFA, SAPS II, and PRESERVE may not reliably reflect outcomes across ARDS subtypes. Subphenotyping ARDS based on its underlying cause can enhance risk assessment and may support more individualized clinical management in this critically ill population.

## Data Availability

The datasets are not publicly available due to data sharing protocols but are available from the corresponding author on reasonable request.
