# Supplementary material for: Effects of the primary lung infection on outcomes in patients with severe ARDS treated with ECMO: a retrospective analysis
Source: Front Med (Lausanne). 2025 Nov 5;12:1662239. doi: 10.3389/fmed.2025.1662239 (PMC12626973; doi:10.3389/fmed.2025.1662239)
Supplement: Supplementary file 1 [file Data_Sheet_1.pdf]

Supplementary Material

1 Supplementary Tables

	Non-Covid ARDS	Covid-19 ARDS	p
Number of patients	N=44	N=48	
Days in hospital before ECMO	8.3 (9.3)	10.9 (5.9)	**0.005**
Days intubated before ECMO	4.8 (6.5)	5.7 (5)	0.129
SAPS II at ICU admission	47.7 (15.0)	40.6 (9.7)	**0.013**
SOFA at ICU admission	15.5 (3.3)	12.6 (3.0)	**<0.001**
SOFA at ECMO-start	15.7 (3.1)<br>(missing N=1)	13.7 (2.6)	**0.003**
SOFA 5 days after ECMO-start	14.4 (3.80)	14.0 (3.41)	0.62
SOFA at discharge	5.8 (5.67)	8.6 (3.54)	**0.048**
Lung Injury Score at ECMO-start	3.25 (0.51)	3.54 (0.32)<br>(missing N=1)	**0.002**
PRESET at ECMO-start	6.99 (2.76)<br>(missing N=4)	5.99 (2.04)<br>(missing N=3)	0.076
RESP at ECMO-start	0.538 (2.42)<br>(missing N=4)	0.354 (2.47)	0.931
PRESERVE at ECMO-start	3.73 (1.63)<br>4 (median)<br>(missing N=4)	2.73 (1.38)<br>3 (median)	**0.004**
Modified EOLIA-criteria fulfilled	N=20 (45.5%)	N=25 (52.1%)	0.67
paO_2 (kPa) at ECMO-start	10.2 (7.02)	8.6 (1.92)	0.621
Horowitz at ECMO-start	12.6 (8.37)	9.15 (2.31)	0.339
Compliance (ml/mbar) at ECMO-start	24.3 (9.8)	23.1 (7.24)	0.866
Infectious source<br>bacterial<br>viral<br>No detection	 N=14 (31.8%)<br>N=15 (34.1%)<br>N=15 (34.1%)	 <br>N=48 (100%)	**<0.001**
Co-infection	N=32 (73%)	N=44 (92%)	**0.034**
Platelets at ECMO-start (Gpt/l)	187 (138)	233 (116))	**0.026**
Fibrinogen at ICU admission (g/l)	6.2 (1.9)	6.8 (2.1)	0.088
Fibrinogen at ECMO-start (g/l)	5.8 (2.0)	7.2 (2.0)	**<0.001**
Fibrinogen max (g/l)	7.7 (1.7)	8.7 (1.6)	**0.002**
DD at ICU admission (ng/ml)	3490 (1030)	5910 (5300)	0.355
DD at ECMO-start (ng/ml)	3570 (883)	5730 (5160)	0.59
DD max (ng/ml)	3470 (1090)	14400 (8940)	**<0.001**
PCT at ICU admission (ng/ml)	46.8 (91.4)	4.6 (8.2)	**0.006**
PCT at ECMO-start (ng/ml)	44.8 (91.5)	5.83 (8.74)	0.192
PCT max (ng/ml)	51.7 (91.4	15.0 (17.8)	0.277
CRP at ECMO-start (mg/l)	237 (139)	235 (102)	0.913
CRP max	355 (135)	349 (94.0)	0.997
Crea at ECMO-start (μ mol/l)	171 (124)	135 (86.8)	0.215
Urea at ECMO-start (mmol/l)	13.8 (7.61)	14.0 (7.56)	0.793
Bili max (μ mol/l)	77.4 (111)	119 (113)	**0.015**
GGT max (μ mol/s*l)	9.8 (10.2)	17.6 (16.0)	**0.007**
Thromboses<br>DVT<br>PE	 N=0<br>N=3 (7%)	 N=14 (29%)<br>N=17 (35%)	 **0.004**<br>**0.002**

Non	N=40 (91%)	N=29 (60%)	**0.002**

Table S3: *Comparison of ARDS course parameters between the two infectious ARDS groups*.
SAPS II: simplified acute physiology score II; SOFA: sepsis-related organ failure assessment score; PRESET: prediction of survival with the ECMO therapy score; RESP: respiratory ECMO survival prediction score; PRESERVE: prediction of death for severe ARDS with the vvECMO score; DD: D-dimer; PCT: procalcitonin; CRP: C-reactive protein; Crea: creatinine; Bili: total bilirubin; GGT: serum gamma-glutamyl transferase; DVT: deep vein thrombosis; PE: pulmonary embolism; unless otherwise noted, mean (SD). Unpaired two-sample Wilcoxon or chi-square test.

	Non-Covid ARDS			Covid-19 ARDS		
	Not survived	survived	p	Not survived	survived	p
Number of patients	N=24 (54.5%)	N=20 (45.5%)		N=33 (68.8%)	N=15 (31.3%)	
Days in hospital before ECMO	10.4 (10.2)	5.9 (7.6)	**0.029**	10.7 (6.2)	11.2 (5.4)	0.824
Days intubated before ECMO	5.3 (6.6)	4.2 (6.5)	0.098	5.9 (4.6)	5.5 (5.9)	0.554
SAPS II at ICU admission	50.6 (14.3)	44.2 (15.6)	0.243	43.6 (9.7)	34.1 (5.6)	**0.001**
SOFA at ICU admission	15.6 (3.2)	15.3 (3.4)	0.678	13.2 (3)	11.3 (2.7)	0.065
SOFA at ECMO-start	16 (2.8)	15.3 (3.3)	0.532	14.2 (2.8)	12.5 (1.9)	0.071
SOFA 5d after ECMO-start	13.8 (3.89)	14.9 (3.74)	0.502	15.4 (3.20)	11.4 (2.06)	**<0.001**
Lung Injury Score at ECMO-start	3.28 (0.569)	3.22 (0.445)	0.45	3.56 (0.33)	3.51 (0.28)	0.38
PRESET at ECMO-start	7.74 (2.31)	6.16 (3.02)	0.062	6.36 (2.16)	5.08 (1.38)	0.053
RESP at ECMO-start	-0.100 (1.94)	1.21 (2.72)	0.059	-0.03 (2.70)	1.20 (1.66)	0.141
PRESERVE at ECMO-start	4.38 (1.43)<br>4 (median)	3.00 (1.56)<br>3 (median)	**0.01**	3.12 (1.24)<br>3 (median)	1.87 (1.30)<br>2 (median)	**0.005**
Modif. EOLIA fulfilled	N=10 (41.7%)	N=10 (50%)	0.804	N=16 (48.5%)	N=9 (60%)	0.668
paO2 (kPa) at ECMO-start	11.3 (9.23)	8.86 (2.56)	0.906	8.67 (2.16)	8.44 (1.21)	1
Horowitz at ECMO-start	12.7 (9.50)	12.4 (7.11)	0.874	8.92 (2.20)	9.70 (2.56)	0.421
Compliance (ml/mbar) at ECMO-start	25.0 (11.3)	23.5 (8.11)	0.824	23.3 (7.62)	22.5 (6.47)	0.863
Infectious source<br>bacterial<br>viral<br>No detection	 <br>N=5 (21%)<br>N=10 (42%)<br>N=9 (38%)	 <br>N=9 (45%)<br>N=5 (25%)<br>N=6 (30%)		 <br>N=0<br>N=33 (68.8%)<br>N=0	 <br>N=0<br>N=15 (31%)<br>N=0	
Co-infection	N=18 (75%)	N=14 (70%)	0.975	N=31 (94%)	N=13 (87%)	0.778
Platelets at ECMO-start (Gpt/l)	165 (120)	213 (156)	0.334	201 (100)	302 (120)	0.008
Fibrinogen at ICU admission (g/l)	6.36 (2.17)	6.08 (1.66)	0.654	6.69 (2.02)	6.98 (2.24)	0.725
Fibrinogen at ECMO-start (g/l)	5.93 (1.91)	5.56 (2.19)	0.649	6.96 (2.08)	7.83 (1.71)	0.41
Fibrinogen max (g/l)	7.46 (1.97)	7.90 (1.21)	0.646	8.57 (1.70)	9.07 (1.41)	0.411
DD at ICU admission (ng/ml)	3340 (1220)	3680 (786)	0.653	6330 (5780)	4930 (3960)	0.7

DD at ECMO-start (ng/ml)	3680 (914)	3420 (901)	0.53	6340 (5810)	4280 (2810)	0.492
DD max (ng/ml)	3590 (1140)	3350 (1050)	0.5	12700 (6700)	17900 (12100)	0.185
PCT at ICU-admission (ng/ml)	27.2 (48.9)	70.3 (122)	0.604	4.84 (8.02)	4.20 (8.79)	0.317
PCT at ECMO-start (ng/ml)	25.5 (49.3)	67.9 (122)	0.646	5.53 (8.11)	6.48 (10.3)	0.965
PCT Max (ng/ml)	31.8 (48.2)	75.6 (122)	0.637	14.6 (15.8)	15.9 (22.3)	0.512
CRP at ECMO-start (mg/l)	229 (116)	247 (164)	0.843	223 (100)	262 (103)	0.354
CRP max	342 (98.3)	370 (171)	1	344 (87.5)	361 (110)	0.66
Crea at ECMO-start (umol/l)	163 (129)	181 (119)	0.697	146 (89.1)	109 (78.1)	0.057
Urea at ECMO-start (mmol/l)	13.9 (7.62)	13.6 (7.80)	0.85	15.6 (8.18)	10.4 (4.40)	**0.018**
Bili max (μ mol/l)	106 (142)	43.2 (36.4)	0.107	145 (116)	61.2 (82.3)	**0.002**
GGT max (μ mol/s*l)	8.58 (9.20)	11.2 (11.4)	0.358	16.8 (17.3)	19.4 (13.0)	0.225
Thrombosis						
DVT	N=0	N=0		N=12 (36%)	N=2 (13%)	**0.013**
PE	N=1 (4%)	N=2 (10%)	0.87	N=13 (39%)	N=4 (27%)	0.597
Non	N=22 (92%)	N=18 (90%)	1	N=20 (61%)	N=9 (60%)	1

Table S4: *Comparison of the ARDS course between deceased and surviving patients in both ARDS groups*.
SAPS II: simplified acute physiology score II; SOFA: sepsis-related organ failure assessment score; PRESET: prediction of survival with the ECMO therapy score; RESP: respiratory ECMO survival prediction score; PRESERVE: prediction of death for severe ARDS with the vvECMO score; DD: D-dimer; PCT: procalcitonin; CRP: C-reactive protein; Crea: creatinine; Bili: total bilirubin; GGT: serum gamma-glutamyl transferase; unless otherwise noted, mean (SD). Unpaired two-sample Wilcoxon or chi-square test.

	Non-Covid ARDS	Covid-19 ARDS	p
Number of patients	N=44	N=48	
Proning	N=36 (81.8%)	N=42 (87.5%)	0.64
Number of pronings	4 (3.8)	6 (4.1)	**0.007**
Pronings before ECMO	2 (1.8)	3 (2.8)	**0.011**
iNO therapy	N=13 (29.5%)	N=30 (62.5%)	**0.003**
Tracheostomy	N=20 (45.5%)	N=27 (56.3%)	0.409
Days intubated till tracheostomy	15.8 (7)	10.9 (6.1)	**0.018**
ECMO BF max (l/min)	4.2 (1.1)	5.1 (2.4)	**0.01**
Sweep max (l/min)	5.1 (2)	7 (2.5)	**<0.001**
Ppeak 24 h after ECMO start (mbar)	25.7 (3.5)	28.0 (2.8)	**0.003**
Ppeak before ECMO-end (mbar)	21.5 (4.5)	26.6 (3.2)	**0.006**
Ppeak 24 h after ECMO-end (mbar)	22.7 (4.5)	28.3 (6.2)	**0.026**
PEEP 24 h after ECMO start (mbar)	11.3 (3.8)	13.8 (1.5)	**0.002**
PEEP before ECMO end (mbar)	10.1 (2.2)	10.9 (1.6)	0.338
PEEP 24 h after ECMO-end (mbar)	9.4 (2.5)	10.3 (2.2)	0.226
Driving pressure 24 h after ECMO start (mbar)	14.7 (4.9)	14.0 (2.8)	0.835
Driving pressure before ECMO-end (mbar)	16.5 (4.4)	14.3 (4.5)	0.882
Driving Pressure 24 h after ECMO-end (mbar)	13.0 (2.5)	13.5 (4.5)	1
VT (ml/kg IBW) before ECMO	5.96 (1.45)	6.10 (1.44)	0.704
VT (ml/kg IBW) 24 h after ECMO start	4.06 (1.55)	4.86 (1.50)	**0.035**
Steroids			
dexamethasone	N=0	N=44 (92%)	**<0.001**
prednisolone	N=18 (41%)	N=22 (46%)	0.791

Other	N=6 (14%)	N=10 (21%)	0.526
New CRRT	N=25 (56.8%)	N=22 (45.8%)	0.399
RBCs	14.9 (13.5)	13.2 (10.1)	0.805
RBCs/day stay	0.841 (0.991)	0.835 (0.677)	0.306
FFPs	6.93 (13.7)	3.02 (4.78)	0.39

Table S5: *Comparison of ARDS therapy between the two infectious ARDS groups*.
iNO: inhalative nitrous oxide; BF: blood flow; Ppeak: peak inspiratory pressure; PEEP: positive end-expiratory pressure; VT: tidal volume; IBW: ideal body weight; CRRT: continuous renal replacement therapy; RBC: packed red blood cells; FFP: fresh frozen plasma. Unless otherwise noted, the data are presented as the means (SDs). Unpaired two-sample Wilcoxon or chi-square test.

	Non-Covid ARDS			Covid-19 ARDS		
	Not survived	survived	p	Not survived	survived	p
Number of patients	N=24 (54%)	N=20 (46%)		N=33 (68.8%)	N=15 (31%)	
Proning	N=19 (79%)	N= 17 (85%)	0.915	N=29 (88%)	N=13 (87%)	1
Number of pronings	4.74 (4.71)	3.18 (2.19)	0.403	6 (3.38)	6.23 (5.49)	0.623
Pronings before ECMO	2.47 (2.09)	0.76 (0.66)	**0.003**	3.34 (2.94)	2.77 (2.62)	0.6
NO therapy	N= 8 (33%)	N=5 (25%)	0.786	N=21 (64%)	N=9 (60%)	1
Tracheostomy	N=7 (29%)	N=13 (65%)	0.038	N=14 (42%)	N=13 (87%)	**0.011**
Days intubated till tracheostomy	16.0 (6.30)	15.6 (7.63)	0.937	9.29 (4.73)	12.7 (7.11)	0.214
ECMO BF max (l/min)	4.57 (0.864)	3.74 (1.17)	**0.013**	5.53 (2.68)	4.16 (0.968)	**<0.001**
Sweep max (l/min)	5.80 (1.94)	4.27 (1.87)	**0.008**	7.79 (2.11)	5.17 (2.37)	**<0.001**
Ppeak 24 h after ECMO start (mbar)	25.8 (3.46)	25.7 (3.63)	0.865	28.2 (2.58)	27.5 (3.27)	0.428
Ppeak before ECMO-end (mbar)	24.0	21.4 (4.56)	0.865	24.0	26.9 (3.31)	0.695
Ppeak 24 h after ECMO-end (mbar)	24.0	22.6 (4.57)	0.714	28.0	28.3 (6.74)	1
PEEP 24 h after ECMO start (mbar)	11.0 (3.58)	11.6 (4.04)	0.369	14.1 (1.18)	13.2 (1.93)	0.103
PEEP before ECMO-end (mbar)	9.00 (1.41)	10.2 (2.27)	0.42	11.0	10.9 (1.62)	0.823
PEEP 24 h after ECMO-end (mbar)	7.0	9.47 (2.46)	0.285	12.0	10.2 (2.26)	0.404
Driving pressure 24 h after ECMO-start (mbar)	14.9 (5.02)	14.5 (4.79)	0.838	13.8 (2.64)	14.3 (3.20)	0.455
VT (ml/kg IBW) before ECMO	5.91 (1.48)	6.02 (1.46)	0.927	5.95 (1.41)	6.48 (1.50)	0.341
VT (ml/kg IBW) 24 h after ECMO-start	3.40 (1.53)	4.76 (1.25)	**0.006**	4.59 (1.52)	5.40 (1.33)	0.096
Steroids						
dexamethasone	N=0	N=0		N=30 (91%)	N=14 (93%)	1
prednisolone	N=14 (58%)	N=4 (20%)	**0.023**	N=14 (42%)	N=8 (53%)	0.696
other	N=6 (25%)	N=0	**0.049**	N=9 (27%)	N=1 (7%)	0.213
New CRRT	N=14 (58%)	N=11 (55.0)	1	N=19 (58%)	N=3 (20%)	**0.035**
RBCs	14.8 (14.8)	15.1 (12.1)	0.697	12.6 (9.61)	14.5 (11.4)	0.632

RBCs/day stay	1.18 (1.23)	0.430 (0.230)	**0.023**	0.970 (0.728)	0.539 (0.437)	**0.03**
FFPs	9.04 (16.8)	4.40 (8.47)	0.158	4.30 (5.29)	0.200 (0.561)	**0.009**

Table S6: *Comparison of ARDS therapy between deceased and surviving patients in both ARDS groups.*
iNO: inhalative nitrous oxide; BF: blood flow; Ppeak: peak inspiratory pressure; PEEP: positive end-expiratory pressure; VT: tidal volume; IBW: ideal body weight; CRRT: continuous renal replacement therapy; RBC: packed red blood cells; FFP: fresh frozen plasma. Unless otherwise noted, the data are presented as the means (SDs). Unpaired two-sample Wilcoxon or chi-square test.
